# Vonoprazan-based triple and dual therapy versus bismuth-based quadruple therapy for *Helicobacter pylori* infection in China: a three-arm, randomised clinical trial protocol

**DOI:** 10.1186/s12876-023-02872-7

**Published:** 2023-07-07

**Authors:** ShaoWei Han, ZiJie Deng, KaShing Cheung, Tao Lyu, PuiLing Chan, Ying Li, Li Ni, XiaPeng Luo, Kuan Li

**Affiliations:** 1 Shantou University Medical College, Shantou, China; 2 Department of Pharmacy, University of Hong Kong-Shenzhen Hospital, Shenzhen, China; 3 Department of Medicine, University of Hong Kong-Shenzhen Hospital, Shenzhen, China; 4 Department of Medicine, School of Clinical Medicine, Li Ka Shing Faculty of Medicine, University of Hong Kong, Hong Kong, China; 5 Department of Pharmacy, Shenzhen People’s Hospital, Second Clinical Medical College, Jinan University, First Affiliated Hospital, Southern University of Science and Technology, Shenzhen, China

**Keywords:** *Helicobacter pylori*, ^13^C-urea breath test, Vonoprazan, Proton pump inhibitor-based regimen

## Abstract

**Background:**

*Helicobacter pylori* infection and associated diseases are a growing global public health issue. *H. pylori* infection is the major cause of gastric cancer, over 90% of duodenal ulcers, and over 70% of gastric ulcers. The infection rate of *H. pylori* is approximately 50%, and approximately 50% of new cases of gastric cancer worldwide occur in China. Bismuth (BI)-based quadruple therapy is recommended as the first-line treatment for *H. pylori* in China. Vonoprazan (VPZ), a new potassium-competitive acid blocker that can inhibit gastric acid secretion more effectively than proton pump inhibitors (PPIs), has been combined with antibiotics to effectively eradicate *H. pylori*. In this study, we compared the efficacy and safety of two VPZ-based therapies with that of BI-based therapy for *H. pylori* treatment.

**Methods:**

A three-armed randomised controlled trial (RCT) is being conducted in Shenzhen, with 327 participants recruited from the Gastroenterology Clinic of the University of Hong Kong-Shenzhen Hospital. Patients were diagnosed with *H. pylori* infection based on a positive ^13^C-urea breath test (UBT). Patients are kept naïve to their treatment and are randomly assigned in a 1:1:1 ratio to either VPZ-based triple, VPZ-based dual, or BI-based quadruple therapy for 14 days. All groups are subjected to follow-up evaluations of safety, adverse drug reactions, and clinical variables in the first, second, and fourth weeks after treatment. Successful eradication is confirmed by a negative ^13^C-UBT six weeks after treatment. If initial treatment fails, (1) those patients are turned to another regimen, or (2) a drug resistance test is conducted, after which an individualised treatment regimen shall be prescribed according to antimicrobial susceptibility testing. The resulting data will be evaluated using intention-treat and a per-protocol analysis.

**Discussion:**

This study is the a RCT aims to evaluate the efficacy and safety of 14-day VPZ-based triple and dual therapies in comparison with BI-based quadruple therapy. The outcomes of this study may allow treatment recommendations and update drug instructions in China.

**Trial registration:**

Chinese Clinical Trial Registry (No. ChiCTR2200056375). Registered on February 4, 2022, https://www.chictr.org.cn/showproj.aspx?proj=141314

## Background

*Helicobacter pylori* (*H. pylori*) is one of the most prevalent bacterial pathogens in the world. *H. pylori* is closely related to upper gastrointestinal diseases, such as gastrointestinal ulcers, gastric mucosa-associated lymphoid tissue lymphoma, and gastric cancer [1–3]. Approximately 50% of new cases of gastric cancer worldwide occur in China [4]. Treating *H. pylori* infection is an effective way to prevent or cure associated diseases [5–7].

In the past, proton pump inhibitor (PPI)-based triple therapy was the recommended regimen for the first-line treatment of *H. pylori* infection. The regimen consists of a PPI plus amoxicillin (AMO) and either clarithromycin (CAM) or metronidazole (MNZ) in areas of low antibiotic resistance [8, 9]. However, owing to the increase in CAM and MNZ resistance rates in recent years, the eradication rate of PPI-based triple therapy has decreased [10, 11]. In China, the drug resistance rates of *H. pylori* to MNZ and CAM are 52.6% and 63.4%, respectively [12]. As a result, bismuth (BI)-containing quadruple therapy has been recommended as an alternative first-line treatment [13–15]. This treatment regimen has increased the eradication rate of resistant *H. pylori* strains by 30–40% [16]. However, antibiotic resistance continues to pose a significant challenge to China's *H. pylori* treatment regimens, necessitating the development of novel treatment strategies to tackle this issue.

Vonoprazan (VPZ), a novel potassium-competitive acid blocker, has demonstrated superior inhibition of gastric acid secretion through H^+^-K^+^ ATPase compared to proton pump inhibitors (PPIs) [17–19]. In a study, after seven days of treatment with 20 mg VPZ twice daily, pH levels of ≥ 4 and ≥ 5 were maintained for the entire duration in 100% and 99% of cases, respectively [18]. VPZ has been approved in Japan for the indications of gastric and duodenal ulcers, reflux esophagitis, and as an adjuvant therapy for *H. pylori* infection. Several meta-analyses have shown that VPZ-based dual or triple therapy outperforms PPI-based regimens in terms of *H. pylori* eradication rates [20–22]. While VPZ was officially approved for marketing in China on December 18, 2019, its current approved indication is limited to reflux esophagitis, and its use for *H. pylori* infection is considered off-label in China. Recent single-center randomized controlled trials (RCTs) conducted in China have confirmed the efficacy and safety of VPZ-based triple therapy [23, 24], providing promising results in the Chinese population. However, larger and multicenter RCTs are still needed to support its indication approval for *H. pylori* infection in China.

This study aims to investigate the efficacy and safety of vonoprazan (VPZ)-based therapy for treating *Helicobacter pylori* (*H. pylori*) infection in China. We propose a three-arm randomized controlled trial (RCT) in which treatment-naive adult patients with *H. pylori* infection will be assigned to receive one of the following regimens: VPZ and amoxicillin (AMO) therapy (VA-dual), VPZ, AMO, and clarithromycin (CAM) therapy (VAC-triple), or bismuth-based quadruple therapy. The treatment duration for all arms will be 14 days, and the allocation will follow a 1:1:1 ratio. Through this RCT, we aim to obtain robust clinical evidence on the efficacy of VPZ-based therapy in Chinese patients, including its effectiveness in different *H. pylori* resistance scenarios. The findings from this study will not only contribute to the development of improved *H. pylori* eradication therapies but also have the potential to reduce the reliance on antibiotics and mitigate the risk of gastric cancer in China.

## Methods

### Study design

**Fig. 1:**
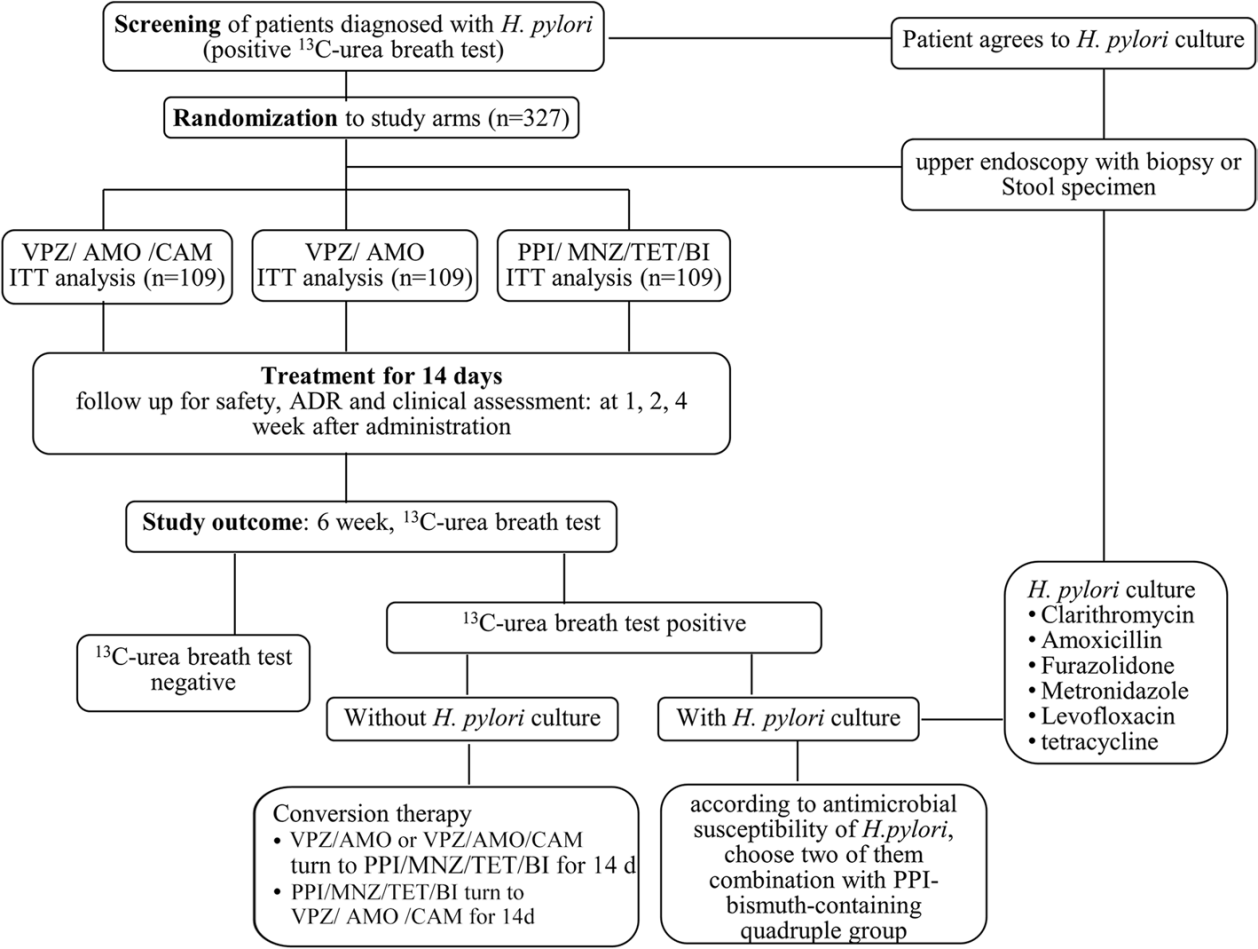
Flow chart of the protocol

The RCT comprising this study aims to compare the efficacy and safety of VA-dual, VAC-triple, and PPI-BI-based quadruple therapies in the treatment of *H. pylori* infection. The flow chart of the Standard Protocol for Randomised Trials is presented in Fig. 1. The study is ongoing between 1 May 2022 and 31 December 2023 at the University of Hong Kong-Shenzhen Hospital, China.

### Study setting and participants

All phases of the study are conducted at the University of Hong Kong-Shenzhen Hospital, which was founded in 2012. In 2019, the hospital's outpatient clinical services reported 1.52 million visits and 65,856 inpatients discharged. Participants are recruited from the gastroenterology clinic at the University of Hong Kong-Shenzhen Hospital, which currently discharges approximately 60,000 outpatients, including patients with *H. pylori* infection. The inclusion and exclusion criteria are summarised in Table 1.

**Table 1:** Inclusion and Exclusion Criteria

**Inclusion Criteria**	
1	Patients were eligible if they were aged 18–75 years and had confirmed *H.pylori* infection by ^13^C-UBT in the outpatient department of ShenZhen Hospital, University of Hong Kong
2	^13^C-UBT (≥ 4) positive can be diagnosed as *H.pylori* infection
**Exclusion Criteria**	
1	History of receiving *H. pylori* eradication therapy
2	Allergy to any of the study drugs
3	History of gastric surgery
4	Antibiotics, bismuth or other drugs that may affect *H.pylori* (including traditional chinese medicine or probiotics) have been used in the past 4 weeks
5	Acid suppressants used in the past 2 weeks
6	Pregnant or breastfeeding
7	Patients treated with clopidogrel were excluded

### Recruitment

Patients who have tested positive for ^13^C-UBT in the outpatient department of Shenzhen Hospital, University of Hong Kong, will be screened by the researchers. Those who meet the inclusion and exclusion criteria will be invited to participate in the study and will be asked to obtain their informed consent. The researchers and participants will each retain a copy of the informed consent document. Recruitment posters for the study are concurrently displayed in the hospital.

### Sample size

In previous studies, eradication rates of VPZ-based regimens for *H. pylori* treatment were between 94 and 91% [25]. We assumed that *H. pylori* eradication rates were 88% for VA-dual, 90% for VAC-triple, and 80% for BI-based quadruple therapies. Non-inferiority design was used in this study and represents approximately 10% of non-inferiority anti-infection trials. Assuming a power of 80%, a one-sided alpha value of 0.025 and follow-up loss of 15% participants are randomly assigned one of the three tested therapies in a 1:1:1 ratio. The non-inferiority trial requires a sample size of 327 patients (109 per treatment group).

### Randomised treatment allocation

Based on the allocation ratio of 1:1:1, 327 eligible patients will be randomly assigned VA-dual, VAC-triple, or PPI- BI-based quadruple therapy. VA-dual consists of 20 mg of VPZ and 1,000 mg of AMO thrice daily. VAC-triple consists of 20 mg of VPZ, 1,000 mg of AMO, and 500 mg of CAM twice daily. PPI-BI-based quadruple therapy consists of 20 mg of esomeprazole (ESO) twice daily, 500 mg of tetracycline (TET) thrice daily, 400 mg of MNZ four times daily and 220 mg of BI twice daily.

Eligible patients that agreed to an initial *H. pylori* culture will undergo either an upper endoscopy with biopsy or collect a stool specimen before undergoing *H. pylori* eradication therapy. The results from the *H. pylori* culture and subsequent antimicrobial susceptibility testing will guide the choice of antibiotics of “rescue” therapy if the ^13^C-urea breath test is still positive after the eradication therapy. Codes of the treatment plan are generated randomly by a computer. All codes are put into separate envelopes, which are kept by the research assistant. After completing the relevant examination, each patient is given an envelope. With informed consent of patients, a research assistant assists the researcher in opening each envelope and determining the treatment plan per patient.

### Bacterial culture and drug sensitivity test

Gastric biopsy specimens are stored immediately in a brain heart infusion broth (Oxoid, Dardilly, France) with 5% glycerin, and stool specimens are stored in the MGIEasy Stool Sample Collection kit (MGI Tech Co., Ltd., ShenZhen, China). These specimens were then sent to the Hangzhou Zhiyuan Medical Laboratory for antibiotic susceptibility testing. *H. pylori* was isolated using Gram staining and enzyme activity tests [26, 27]. The isolated strains of *H. pylori* will be subjected to a drug sensitivity culture of six antibiotics (CAM, levofloxacin, MNZ, AMO, TET, and furazolidone) [27].

### Data collection and measures

Telephonic follow-up evaluations will be performed in the first, second, and fourth weeks after treatment to assess patient compliance and other treatment outcomes. The patient follow-up form used for this evaluation is presented in Table 2. A ^13^C- urea breath test (^13^C-UBT; Headway Bio-Sci & Tech Co, Ltd, Shenzhen, China) will be performed at the beginning of week 6 after treatment.

**Table 2:** Times and events schedule used in patient follow-up evaluations

Name	□	ID		
Age	□	Sex	□M	□F
Initial treatment group		□VA-dual		
		□VAC-triple		
		□ PPI-bismuth-containing quadruple		
Follow-up period		**week 1**	**week 2**	**week 4**
ADRs		□	□	□
Nervous system disorders		□	□	□
Dizziness		□	□	□
Dysgeusia		□	□	□
Hypogeusia		□	□	□
Gastrointestinal disorders		□	□	□
Abdominal discomfort		□	□	□
Abdominal distension		□	□	□
Constipation		□	□	□
Diarrhoea		□	□	□
Faeces hard		□	□	□
Nausea		□	□	□
Paresthesia oral		□	□	□
Faeces soft		□	□	□
Skin and subcutaneous tissue disorders		□	□	□
Drug Eruption		□	□	□
Eczema		□	□	□
Rash		□	□	□
Urticaria		□	□	□
Other		□	□	□
^13^C- urea breath test after 6 week		□ < 4	□ ≥ 4	
Initial treatment group treatment failure				
According to drug sensitivity results (choose two of them)	□ Clarithromycin			
	□ Amoxicillin			
	□ Furazolidone			
	□ Metronidazole			
	□ Levofloxacin			
	□ Tetracycline			
None culture and drug sensitivity results	□ VA-dual or VAC-triple turn to PPI-bismuth-containing quadruple			
	□ PI-bismuth-containing quadruple turn to VAC-triple			

### Study outcome

The goal of this study is to compare the *H. pylori* eradication rate of the two VPZ-based groups with that of the PPI-BI-based quadruple group. The results of ^13^C-UBT (Headway Bio-Sci & Tech Co, Ltd, Shenzhen, China) will be used to evaluate eradication success. Eradication success is defined as (Delta Over Baseline, DOB < 4). Before ^13^C-UBT, patients are not permitted to take PPIs or antibiotics. Those with positive results (DOB ≥ 4) are considered to exhibit failed *H. pylori* eradication. The success of this will be determined using intention-to-treat (ITT) and per-protocol (PP) analysis. The ITT analysis includes all randomised patients. In the ITT analysis, patients who lost their follow-up forms or did not undergo ^13^C-UBT will be grouped under ‘treatment failure’. The PP analysis includes patients with a drug compliance over 80% (10 days) who underwent ^13^C-UBT.

For the patients that failed *H. pylori* eradication, a second standardised medication will be administered two months after ^13^C-UBT as the “rescue” therapy. In the presence of treatment failure without antimicrobial susceptibility testing, patients are assigned to receive another regimen (i.e., switched from PPI-BI-based therapy to VAC-triple therapy; or switched from VAC-triple or VA-dual to PPI-BI-based therapy). In the presence of treatment failure with antimicrobial susceptibility testing, an individualized treatment regimen shall be prescribed according to antimicrobial susceptibility testing.

### Monitoring

Researchers will record all adverse drug reactions (ADRs) in patients’ medical records. Patients exhibiting adverse events (AEs) will be subjected to follow-up evaluations. The experiment will be terminated in cases where patients exhibit serious ADRs, and further medical examinations will be arranged. The participants enrolled in the study will be appropriately treated by healthcare providers, as summarised in steps (a) through (d).

- Inform patients in advance of possible adverse reactions during medication.
- Assess the risk of allergic reactions in patients in advance using drugs.
- Assign special researchers responsible for following up on patients' adverse reactions.
- Provide green access to medical treatment for patients exhibiting adverse reactions.

### Data analysis

We will evaluate and compare the results of the non-inferiority trial of the three groups using two-sided 95% CI and hypothesis testing (one-sided μ test). Pearson’s χ2 test and Student’s t-test will be used to analyse differences in categorical and continuous variables between groups, respectively. Except for the test of non-inferiority, all *P* values are two-sided, and *P* values < 0.05 are considered statistically significant.

Once patients are enrolled in the study, they will not be allowed to change from one group to another, but they can terminate the clinical trial at any time. The researchers will keep the source data of each patient, including their signed informed consent, hospital medical records, test results, and other records.

### Ethical issues

This study conforms to the ethical code stated in the Declaration of Helsinki. The research protocols were approved by the Ethics Committee of Shenzhen Hospital of the University of Hong Kong (approval number: [2022] 015).

## Discussion

In China, PPI-BI-based quadruple therapy is currently recommended as the main empiric treatment for *H. pylori* eradication. The Fifth Chinese National Consensus Report on the management of *Helicobacter pylori* infection recommends seven PPI-BI-based quadruple schemes [28]. This study uses PPI, BI, TET, and MNZ therapy, one of these seven treatment options, as the control group. According to a previous study at the University of Hong Kong–Shenzhen Hospital, the drug resistance rate of TET is 0% [29]. Additionally, according to the HOMER study, the eradication success rates of MNZ-resistant strains were 54%, 50%, and 75% at dosages of 800, 1,200, and 1,600 mg/day, respectively [30, 31]. Thus, we optimised the dose of MNZ at 1,600 mg/day.

Given that VPZ is not approved for the treatment of *H. pylori* in China, the off-label use of VPZ for that purpose was registered in the Drug and Therapeutics Committees at the University of Hong Kong-Shen Zhen Hospital. This allows VPZ-based triple and dual therapy in the treatment of patients infected with *H. pylori* within the scope of this study, without violating any relevant laws, regulations, or hospital procedures.

This study aims to conduct a randomized controlled trial (RCT) to compare the efficacy and safety of 14-day VAC-triple and VA-dual therapies with BI-based quadruple therapy for treating *H. pylori* infection. The primary goal is to gather substantial data on the effectiveness of VPZ-based therapy in the Chinese population. The results obtained from this RCT have the potential to inform updated treatment recommendations and drug instructions in China. Additionally, VPZ-based therapy, particularly VPZ-dual therapy, may help reduce unnecessary antibiotic use, aligning with the principles of antibiotic stewardship. It is important to acknowledge that this study has limitations, including its limited sample size and single-center design. To validate our findings, further prospective multicenter studies with larger sample sizes and comprehensive treatment outcome data should be conducted. Through this RCT, we strive to provide robust clinical evidence regarding the efficacy of VPZ-based therapy in Chinese patients, taking into account different *H. pylori* resistance scenarios. The findings from this study not only contribute to the development of improved *H. pylori* eradication therapies but also have the potential to reduce antibiotic reliance and mitigate the risk of gastric cancer in China.

## Data Availability

Data and materials are currently not available, as the trial is still in progress. Future data from this trial can be obtained from the corresponding author upon reasonable request.

## Abbreviations

PPI: Proton pump inhibitor
RCT: Randomised controlled trial
VA: Vonoprazan and amoxicillin
VAC: Vonoprazan, amoxicillin and clarithromycin
ADR: Adverse drug reaction
UBT: Urea breath test
VPZ: Vonoprazan
AMO: Amoxicillin
CAM: Clarithromycin
ESO: Esomeprazole
TET: Tetracycline
MNZ: Metronidazole
BI: Bismuth
ITT: Intention-to-treat
PP: Per-protocol
AEs: Adverse events
